# Health-related quality of life of metastatic prostate cancer patients treated with prostate Radiotherapy

**DOI:** 10.1186/s12885-023-11448-3

**Published:** 2023-10-02

**Authors:** Heba Maged Ayoub, Maha Lotfy Zamzam, Eman Essam Elsemary, Ihab Mohamed Hassanin, Fifi Mostafa Elsayed

**Affiliations:** Clinical Oncology Department, Faculty of Medicine, Suez Canal University, Ismailia, Egypt

**Keywords:** ADT, Cytoreductive, HRQoL, Metastatic prostate cancer, Prostate radiotherapy

## Abstract

**Background:**

The impact of prostate radiotherapy on patient-reported health-related quality of life (HRQoL) significantly influences the outcomes of metastatic prostate cancer. We measured and compared HRQoL of metastatic prostate cancer patients who received cytoreductive prostate radiotherapy.

**Methods:**

Between November 23, 2020, and November 21, 2022, we recruited 70 metastatic prostate cancer patients at the Department of Clinical Oncology at Suez Canal University Hospital. Patients were eligible if they had synchronous and metachronous histopathological confirmed metastatic adenocarcinoma and an Eastern Cooperative Oncology Group performance status ≤ 2. Random allocation was performed for either definitive local radiotherapy concurrent with the standard androgen deprivation therapy (ADT) or to the standard ADT alone. Definitive radiotherapy was delivered conventionally (70 Gy/35 fractions) or through the hypofractionated regimen (55 Gy/20 fractions). Patients completed the comprehensive European Organization for Research and Treatment of Cancer Quality of Life Questionnaire (EORTC QLQ-PR25) at baseline, then at three-month intervals for one year. The primary endpoint was patient-reported HRQoL, with secondary endpoints including toxicity and radiographic progression-free survival (rPFS). Mean HRQoL scores between groups were compared using the independent samples t-test.

**Results:**

We observed clinically significant improvements in urinary and bowel functions between baseline, 3-month, 6-month, and 12-month intervals after receiving definitive prostate radiotherapy. Patients in the radiotherapy group had significantly lower urinary symptoms scores than the control group. However, sexual activity and functioning showed significant deterioration.

**Conclusion:**

Cytoreductive prostate radiotherapy in metastatic prostate cancer patients significantly improved urinary functioning, preserved bowel functioning but was associated with worsening of sexual functioning.

**Trial Registration:**

This trial was registered on (27/04/2023) with pactr.samrc.ac.za, PACTR202305854600529, URL: https://pactr.samrc.ac.za/TrialDisplay.aspx?TrialID=25510.

**Supplementary Information:**

The online version contains supplementary material available at 10.1186/s12885-023-11448-3.

## Introduction

Prostate cancer has been globally expanding placing a high burden on patient-reported health-related quality of life (HRQoL). Recently, prostate cancer has become the second mostly diagnosed male cancer with a rising incidence by 3% annually for the last 5 years. According to SEER data, an estimated 288,300 new prostate cancer patients will be diagnosed in 2023 representing approximately 14% of all new cancer cases [1]. Metastatic disease accounts for 6–8% of newly diagnosed prostate cancer cases [2].

Upfront combination treatments that have been greatly encouraged and adopted in the management of metastatic prostate cancer patients have negatively impacted upon health-related quality of life (HRQoL) [3]. Short-term and long-term HRQoL should be considered before choosing the most tailored treatment for metastatic prostate cancer patients [4].

Prostate-directed radiotherapy is a new standard practice that has shown substantial benefits in delaying disease progression and improving survival outcomes (5, 6). However, combining prostate radiotherapy with the standard androgen-deprivation therapy affects patients’ physical, emotional, sexual, and social health. Ultimately, HRQoL after local radiotherapy depends on the total dose, fractionation regimen and the dose received by the critical organs [7].

Early genitourinary toxicities may include obstructive and irritative symptoms such as frequency, nocturia and urgency. Early gastrointestinal toxicities include diarrhea, rectal bleeding, and abdominal pain. These symptoms typically disappear within 4–8 weeks after finishing prostate radiotherapy. However, some patients might suffer from proctitis with rectal bleeding as a late rectal toxicity. Less commonly patients might suffer hematuria as a late bladder toxicity [8].

Undoubtedly, assessing health-related quality of life facilitates improved treatment outcomes. Focusing on reporting various quality of life domains, including urinary, sexual and bowel functions for each patient is associated with improved survival outcomes [9, 10]. Thus, standardizing HRQoL assessment tools, their scoring and interpretation in metastatic prostate cancer patients receiving prostate radiotherapy is necessary to optimize treatment outcomes and improve patients’ adherence to treatment and health care facilities [9]. In addition to using validated patient-reported outcome measures (PROMs) to evaluate HRQoL, reporting radiotherapy-related adverse events is complementary to assess patients’ tolerability and treatment efficacy. This study aimed to assess the impact of delivering cytoreductive local radiotherapy concurrently with ADT on patient reported HRQoL. We hypothesized that administering cytoreductive prostate radiotherapy to metastatic prostate cancer patients would lead to an improvement in health-related quality of life outcomes.

## Methods

### Study design and conduct

A phase III randomized controlled study was conducted from November 2020 to November 2022 at the Clinical Oncology Department, Suez Canal University Hospital in Ismailia, Egypt. The study aimed to assess the patient-reported HRQoL and toxicity profile in metastatic prostate cancer patients who received local prostate-directed radiotherapy concurrent with ADT. The study protocol was approved by the Research Ethics Committee at the Faculty of Medicine, Suez Canal University prior to its commencement. All patients provided written informed consent before enrollment and had the right to withdraw from the study at any time.

### Patients

This study encompassed eligible patients with either de-novo or metachronous metastatic prostate cancer, defined according to the European Society for Radiotherapy (ESTRO) and the European Organization for Research and Treatment of Cancer (EORTC) criteria for oligometastatic disease [11]. Patients with histologically proven adenocarcinoma and a performance score ≤ 2 according to the Eastern Cooperative Oncology Group (ECOG) performance scale, with or without nodal involvement, low metastatic burden prostate cancer (< 4 bone metastases), or high metastatic burden prostate cancer (≥ 4 bone metastases at least 1 beyond spine or pelvis, ± visceral metastases) according to CHAARETED definition. Patients were excluded if they had a second neoplasm, ECOG performance score > 2, underwent radical prostatectomy, received prior pelvic radiotherapy, or had cerebral metastases. We recruited and enrolled 70 patients at our institution between November 2020 and November 2022. Then, 63 patients were randomly allocated to receive either prostate radiotherapy concurrent with ADT (Prostate RT group) or the standard ADT (control group).

### Randomization and masking

Randomization was conducted using computer generated block randomization with a block size of 4 which consisted of 2 treatments with 2 patients per treatment. Stratification of patients at randomization was not performed, and allocation was not masked due to the nature of the intervention.

### Intervention

All patients with metastatic prostate cancer received the standard ADT (Gonadotropin hormone–releasing hormone agonist ± bicalutamide) or underwent orchiectomy 2 months prior to randomization. ADT was subsequently administered concurrently with radiotherapy and continued thereafter. Local external beam radiotherapy was administered as one of two regimens either: conventional regimen 70 Gy in 35 daily fractions of 2 Gy over 7 weeks, or hypofractionated regimen 55 Gy in 20 daily fractions of 2.75 Gy over 4 weeks. Patients were simulated in supine position with an empty rectum and comfortably full bladder. Pelvic immobilization was maintained using knee support. Planning CT scans were acquired without intravenous contrast. We employed forward planned three-dimensional (3D) conformal radiotherapy with planning target volume (PTV) that encompassed the prostate with a 10 mm margin circumferentially, except posteriorly (6 mm) and the proximal 2 cm of seminal vesicles. Elective pelvic nodal irradiation was not included in the field. Patients were followed-up weekly during radiotherapy, then 1 month after finishing radiotherapy, then every 3 months for a year.

All patients were then requested to complete the European Organization for Research and Treatment of Cancer Quality-of-Life Questionnaire Prostate Module (EORTC-QLQ-PR25) at baseline and then every 3 months throughout the study. Patient reported outcome measures (PROMs) were filled in and registered using paper forms. This module consists of multi-item scales designed to measure prostate cancer-specific health-related quality of life outcomes (Supplementary File. 1). The module comprises 25 questions, all of which should be completed by the patient. We used this questionnaire to assess baseline HRQoL in metastatic prostate cancer patients receiving standard of care and to determine changes in patient-reported HRQoL after prostate radiotherapy [10]. The questions in the prostate cancer module are grouped into symptom scales and functional scales. The symptoms scales assess urinary symptoms (Questions 1–7,9), incontinence aid (Question 8), bowel symptoms (Questions 10–13) and hormonal treatment-related symptoms (Questions 14–19). The functional scales consist of questions related to sexual activity (Questions 20,21) and sexual functioning (Questions 22–25). Scoring of the EORTC QLQ-PR25 was carried out according to the provided EORTC scoring manual (Supplementary File. 2) [10]. The calculated raw score for each scale is linearly transformed into a scale from 0 to 100 points. Higher scores on symptomatic scales indicate greater symptom severity whereas higher scores on functioning scales indicate better levels of functioning.

During treatment, early and late genitourinary and gastrointestinal toxicity symptoms were reported weekly. After treatment, these symptoms were reported every 3 months during regular follow-up visits or when serious adverse events occurred, and these events were graded according to the Radiation Therapy Oncology Group (RTOG) scale. Early toxicity was recorded within the first 90 days post-radiation while late toxicity was recorded after 90 days. The metastatic burden of the disease was assessed through CT and bone scans. Radiographic response after radiation was monitored every 6 months using CT chest and pelvi-abdominal scans and evaluated based on the RECIST 1.1 criteria [12].

### Endpoints

The primary endpoint of this study was patient-reported health-related quality of life (HRQoL). Secondary endpoints included early and late radiation toxicity for the prostate-radiotherapy group and radiographic progression-free survival.

### Statistical analysis

The target sample size was calculated as 50 patients. This provided 80% power (one-sided-α of 0.025). Statistical analysis was conducted using the Statistical Package for Social Science (SPSS), version 28 (IBM Corporation, Armonk, NY, USA). The statistical significance of the difference between mean scores between the prostate radiotherapy group and the control group was tested using independent-sample t test. Friedman’s test was used to compare mean HRQoL scores between baseline and different time points post-radiation for one year. We managed missing data by performing a complete-case analysis, which involved excluding items related to censored or lost follow-up patients. Additionally, we addressed the missing items in the scale through imputation method.

Fisher’s exact test and Pearson’s Chi square test were conducted to test for the significance of association between variables. Radiographic progression-free survival was measured using Cox Regression analysis and the Log-Rank test was used for comparison between study groups. The hazard ratio and its 95% confidence interval were calculated using multivariate Cox regression to determine the association between variables and radiographic progression-free survival. The statistical significance for radiographic progression-free survival was measured using the Wilcoxon signed rank test. Post-hoc survival analysis was conducted to correlate radiographic progression-free survival with the metastatic burden of the disease. Additionally, exploratory subgroup analysis was performed to compare the median radiographic progression-free survival time among different subgroups. Subgroup analyses were pre-specified for the baseline metastatic volume (low vs. high), the response to androgen deprivation (castration sensitive vs. resistant) and the received radiotherapy regimen (conventional 70 Gy vs. hypofractionated 55 Gy). All statistical tests were two-sided, and *p*-values of < 0.05 were considered statistically significant.

## Results

**Fig. 1:**
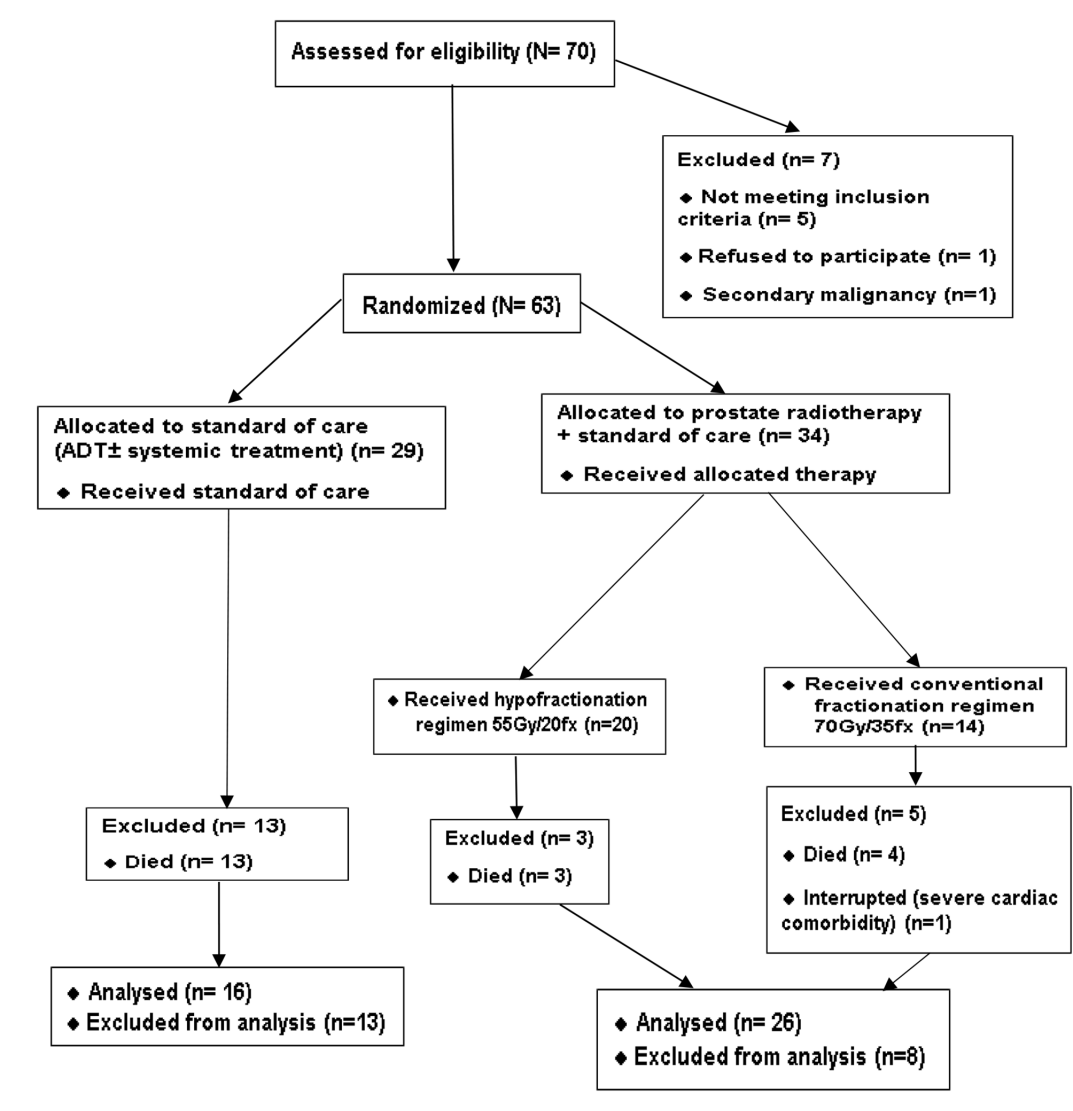
CONSORT flow diagram

From November 23, 2020, to November 21, 2022, a total of 63 patients were enrolled and randomly assigned to either the control group receiving standard ADT (n = 29) or the radiotherapy group receiving standard ADT and radiotherapy (n = 34). Among the radiotherapy group, 20 patients received the hypofractionated radiation schedule, while the remaining 14 patients received the conventional fractionation schedule. Only one patient in the conventional fractionation group stopped treatment after receiving 11 sessions due to severe cardiac comorbidity (Fig. 1). We used the CONSORT Patient reported outcomes (PROs) extension checklist for reporting PROs.

The study groups were well balanced regarding baseline sociodemographic and clinicopathological characteristics.

The median age of patients was 70 years (IQR 66–75). The most common histopathological variant in both study groups was acinar adenocarcinoma (88.9%). The most frequent histological grade observed was GS 8–10 (47.6%). The most common pathological stages were T3 (38.1%) and N0 (61.9%). The most common site of metastases was bone (69.8%). The radiotherapy group had a significantly higher incidence of nodal positive disease (*p =* 0.035). High-volume metastatic disease was present in 63.5% of patients in both groups, and most patients had synchronous metastatic disease (85.7%). Most patients in both groups had an ECOG performance score of 1 (65.1%). Clinically, most patients suffered frequency (87.3%), urgency (95.2%), difficulty (88.9%), and dysuria (84.1%). Patients in the control group had significantly more skeletal-related events than those in the radiotherapy group (62.1%, *p =* 0.001). The median duration of androgen deprivation therapy administered before randomization was 4.25 months (IQR 2.5-9) for the radiotherapy group and 2.8 months (IQR 1.94-7) for the control group. Most patients received combined GnRH agonist and bicalutamide (71.4%). The majority of patients were castrate sensitive (68.3%), while 31.7% were castrate resistant. Docetaxel was received by 25.4% of patients in both groups, whereas 22.2% received abiraterone acetate. Very few patients received enzalutamide (4.8%) (Table 1).

**Table 1:** Sociodemographic and clinicopathological characteristics among study groups

Variables	Prostate RT group n = 34 (54%)	Control group n = 29 (46%)	*P-*value
Age (years) < 65	10 (29.4%)	4 (13.8%)	0.224 ^f^
> 65	24 (70.6%)	25 (86.2%)	
Median (IQR)	68 (64–72)	73 (68.5–79.5)	
Marital status Married	24 (70.6%)	14 (48.3%)	0.068 ^f^
Single	10 (29.4%)	15 (51.7%)	
Residence Rural	10 (29.4%)	11 (37.9%)	0.478
Urban	24 (70.6%)	18 (62.1%)	
Histopathological variant Acinar adenocarcinoma	32 (94.1%)	24 (82.8%)	0.233 ^f^
Others	2 (5.9%)	5 (17.2%)	
GS ≤ 7	12 (35.3%)	4 (13.8%)	0.081 ^f^
8–10	22 (64.7%)	25 (86.2%)	
T Stage T2	13 (38.2%)	8 (27.6%)	0.371
T ≥ 3	21 (61.8%)	21 (72.4%)	
N Stage N0	17 (50%)	22 (75.9%)	0.035*
N+	17 (50%)	7 (24.1%)	
Site of metastases Bones	23 (67.6%)	21 (72.4%)	0.628 ^f^
Lungs	2 (5.9%)		
> 1 site	9 (26.5%)	8 (27.6%)	
Metastatic burden High metastatic burden	18 (52.9%)	22 (75.9%)	0.060
Low metastatic burden	16 (47.1%)	7 (24.1%)	
Timing of Metastases Synchronous	27 (79.4%)	27 (93.1%)	0.160 ^f^
Metachronous	7 (20.6%)	2 (6.9%)	
Performance status			
≤ 1	25 (73.5%)	17 (58.6%)	0.211
2	9 (26.5%)	12 (41.4%)	
Prior ADT Duration, median (IQR)	4.25 (2.5-9)	2.8 (1.9-8)	0.073
GnRH agonist only	3 (8.8%)	5 (17.2%)	0.453 ^f^
Bicalutamide only	6 (17.6%)	3 (10.3%)	0.488 ^f^
Both	25 (73.5%)	21 (72.4%)	0.921
Response to ADT Castration naïve/sensitive	24 (70.6%)	19 (65.5%)	0.666
Castration resistant	10 (29.4%)	10 (34.5%)	
Docetaxel	7 (20.6%)	9 (31%)	0.342
Abiraterone acetate	10 (29.4%)	4 (13.8%)	0.224 ^f^
Enzalutamide	2 (5.9%)	1 (3.4%)	1.000 ^f^
Palliative RT	9 (26.5%)	25 (86.2%)	< 0.001*
Comorbidities*	22 (64.7%)	19 (65.5%)	0.946

RT: radiotherapy, IQR: Interquartile range, N+: node positive, ADT: Androgen deprivation therapy,

Comorbidities; hypertension in 30 patients, Diabetes Mellitus in 28 patients, Congestive heart failure in 10 patients, CKD in 10 patients and COPD in 4 patients.

GnRH: Gonadotropin releasing hormone SRE: skeletal-related events, GS: Gleason score.

^f^. Fisher’s exact test *. Statistically significant p value.

After a median follow-up duration of 12 months (IQR 10–14), there was a significant improvement in urinary and bowel functions at 3 months, 6 months, and 12 months intervals compared to baseline. However, there was a statistically significant worsening of ADT related symptoms, sexual activity, and sexual functioning at 3 months, 6 months, and 12 months intervals compared to baseline (Table 2).

**Table 2:** Health-related Quality of life scoring of the prostate radiotherapy group over one year

EORTC QLQ-PR25	Mean (SD)	*P-*value
Urinary functions Baseline	76.76 (15.1)	< 0.001*
3 months	15.06 (17.32)	
6 months	12.02 (15.15)	
9 months	12.02 (15.15)	
12 months	11.22 (15.17)	
Incontinence aid Baseline	14.10 (31.51)	0.144
3 months	10.26 (26.28)	
6 months	11.54 (29.73)	
9 months	11.54 (29.73	
12 months	11.54 (29.73)	
Bowel functions Baseline	13.78 (10.26)	< 0.001*
3 months	10.26 (5.92)	
6 months	8.79 (5.74)	
9 months	8.33 (5.78)	
12 months	8.33 (5.78)	
ADT-related symptoms Baseline	57.1 (17.11)	< 0.001*
3 months	60.47 (17.87)	
6 months	63.03 (17.98)	
9 months	64.74 (18.52)	
12 months	64.96 (18.51)	
Sexual activity Baseline	32.05 (18.81)	< 0.001*
3 months	18.27 (14.72)	
6 months	16.34 (14.24)	
9 months	13.78 (10.53)	
12 months	13.46 (10.56)	
Sexual function Baseline	14.10 (12.42)	< 0.001*
3 months	3.53 (5.86)	
6 months	3.53 (5.86)	
9 months	3.53 (5.86)	
12 months	3.53 (5.86)	

*. Statistically significant *p* value

Patients in the prostate radiotherapy group had statistically significantly lower and better scores in urinary symptoms and ADT related symptoms than patients in the control group at 3 months, 6 months, 9 months, and 12 months (*p <* 0.001) (Table 3).

**Table 3:** Comparing health-related quality of life domains scoring between study groups

QLQ-PR25 Variables	Prostate RT group	Control group	*P-*value
	**Mean (SD)**	**Mean (SD)**	
QoL Urinary functions			
Baseline	78.19 (15.11)	77.44 (14.58)	0.844
3 months	18.75 (20.25)	62.19 (15.67)	< 0.001*
6 months	15.19 (17.33)	59.24 (18.20)	< 0.001*
9 months	12.5 (14.96)	61.04 (20.56)	< 0.001*
12 months	11.22 (15.17)	59.29 (22.89)	< 0.001*
QoL Bowel functioning			
Baseline	15.44 (11.26)	8.33 (8.34)	0.707
3 months	12.5 (9.7)	13.58 (14.83)	0.738
6 months	11.02 (8.97)	11.96 (13.02)	0.756
9 months	8.63 (5.78)	13.33 (13.63)	0.108
12 months	15.38 (20.93)	14.08 (17.12)	0.114
QoL Incontinence aid			
Baseline	16.67 (83.34)	31.03 (44.48)	0.147
3 months	13.54 (30.36)	25.93 (38.49)	0.173
6 months	11.83 (29.25)	20.29 (31.37)	0.313
9 months	10.71 (28.77)	21.67 (34.67)	0.239
12 months	11.54 (29.73)	28.21 (38.12)	0.142
QoL ADT symptoms			
Baseline	58.33 (16.08)	68.01 (10.46)	0.006*
3 months	62.33 (17.04)	75.72 (6.19)	< 0.001*
6 months	64.87 (17.36)	77.05 (4.53)	< 0.001*
9 months	65.87 (18.31)	78.61 (4.52)	0.001*
12 months	64.96 (18.51)	79.06 (5.15)	0.001*
QoL Sexual activity			
Baseline	28.92 (18.03)	12.64 (12.32)	< 0.001*
3 months	16.40 (13.96)	9.88 (11.56)	0.058
6 months	14.78 (13.56)	8.7 (12.17)	0.095
9 months	13.1 (10.5)	6.67 (11.34)	0.053
12 months	13.46 (10.56)	7.69 (11)	0.121
QoL Sexual functioning			
Baseline	12.26 (11.83)	2.3 (5.41)	< 0.001*
3 months	2.86 (5.44)	2.47 (4.51)	0.765
6 months	2.96 (5.51)	1.89 (3.57)	0.431
9 months	3.27 (5.71)	0.83 (2.56)	0.081
12 months	3.53 (5.86)	0.64 (2.31)	0.097

*. Statistically significant p value derived from independent samples t-test, SD: standard deviation.

QoL: quality of life, RT: radiotherapy, QLQ: quality of life questionnaire, ADT: Androgen deprivation therapy.

Furthermore, there was no statistically significant difference between patients who received either fractionation regimens in terms of early or late genitourinary and gastrointestinal events. The most observed significant acute GU toxicity in both fractionation arms was cystitis (G2 in 58.8% and G3 + in 32.3%). Acute G2 hematuria developed in 38.2% and G3 + in 32.3% of patients. Acute G2 urinary obstruction occurred in 2.9% and G3 + in 32.3% of patients (Table 4).

Meanwhile, acute proctitis and abdominal pain were the most common acute GI toxicities in both fractionation schedules, with G2 toxicity occurring in 32.3% of patients and G3 + in 14.7%. Acute G2 diarrhea was reported in 8.8% of patients, and G3 + in 3 patients (8.8%). Patients who received the hypofractionation regimen experienced a higher-grade acute diarrhea. In addition, acute anal fissure/fistula was detected in 3 patients (8.8%), with 2 patients developing acute anal fissure and one patient developing acute perianal fistula. Acute G3 + rectal bleeding developed in 3 patients (8.8%) (Table 4).

The most common late GU toxicities observed in both fractionation schedules were late cystitis and bladder spasm, with G2 toxicity occurring in 6.4% of patients and G3 + in 12.9%. Late hematuria was reported as G2 in 3.2% of patients and G3 + in 12.9%. Four patients (12.9%) developed late G3 + urinary obstruction, and all patients in both arms experienced erectile dysfunction.

The most prevalent late GI toxicities in both fractionation schedules were proctitis and abdominal pain, with G2 toxicity occurring in 3.2% of patients and G3 + in 3.2%. Additionally, late rectovesical fistula developed in 1 patient (3.2%) (Table 4).

**Table 4:** Incidence of significant genitourinary and gastrointestinal toxicities in patients receiving prostate radiotherapy

Toxicity	Fractionation type		*P-*value
	**Conventional** **n = 14 (41.2%)**	**Hypofractionation** **n = 20 (58.8%)**	
Acute GU toxicity G2+	12 (85.7%)	19 (95%)	0.555 ^f^
Cystitis, bladder spasm G2	9 (75%)	11 (57.9%)	0.452 ^f^
G3+	3 (25%)	8 (42.1%)	
Hematuria G2	6 (66.7%)	(46.7%)	0.423 ^f^
G3+	3 (33.3%)	8 (53.3%)	
Obstruction G2	0 (0%)	(12.5%)	1.000 ^f^
G3+	4 (100%)	7 (87.5%)	
Acute GI toxicity G2+	7 (50%)	9 (45%)	0.774
Diarrhea G2	3 (100%)		0.100 ^f^
G3+		3 (100%)	
GI upset G2	4 (100%)	1 (25%)	0.143 ^f^
G3+		3 (75%)	
Proctitis, Abdominal pain G2	6 (85.7%)	5 (55.6%)	0.308 ^f^
G3+	1 (14.3%)	4 (44.4%)	
Fissure/ fistula G3+	2 (14.3%)	1 (5%)	0.555 ^f^
Rectal bleeding G3+	2 (14.3%)	1 (5%)	0.555 ^f^
Late GU toxicity G2+	2 (16.7%)	3 (17.6%)	1.000 ^f^
Cystitis, bladder spasm G2	1 (33.3%)	1 (33.3%)	1.000 ^f^
G3+	2 (66.7%)	2 (66.7%)	
Hematuria G2		1 (33.3%)	1.000 ^f^
G3+	2 (100%)	2 (66.7%)	
Obstruction G3+	(16.7%)	2 (11.8%)	1.000 ^f^
Erectile dysfunction	11 (100%)	17 (100%)	
Late GI toxicity G2+	1 (9.1%)	1(5.9%)	1.000 ^f^
Proctitis, Abdominal pain G2		1 (100%)	1.000 ^f^
G3+	1 (100%)		
Fissure/ fistula G3+	1 (9.1%)		0.393 ^f^

^f^. Fisher’s exact test. GU: Genitourinary, GI: Gastrointestinal, G2+: Grade 2 or higher, G3+: Grade 3 or higher

**Fig. 2:**
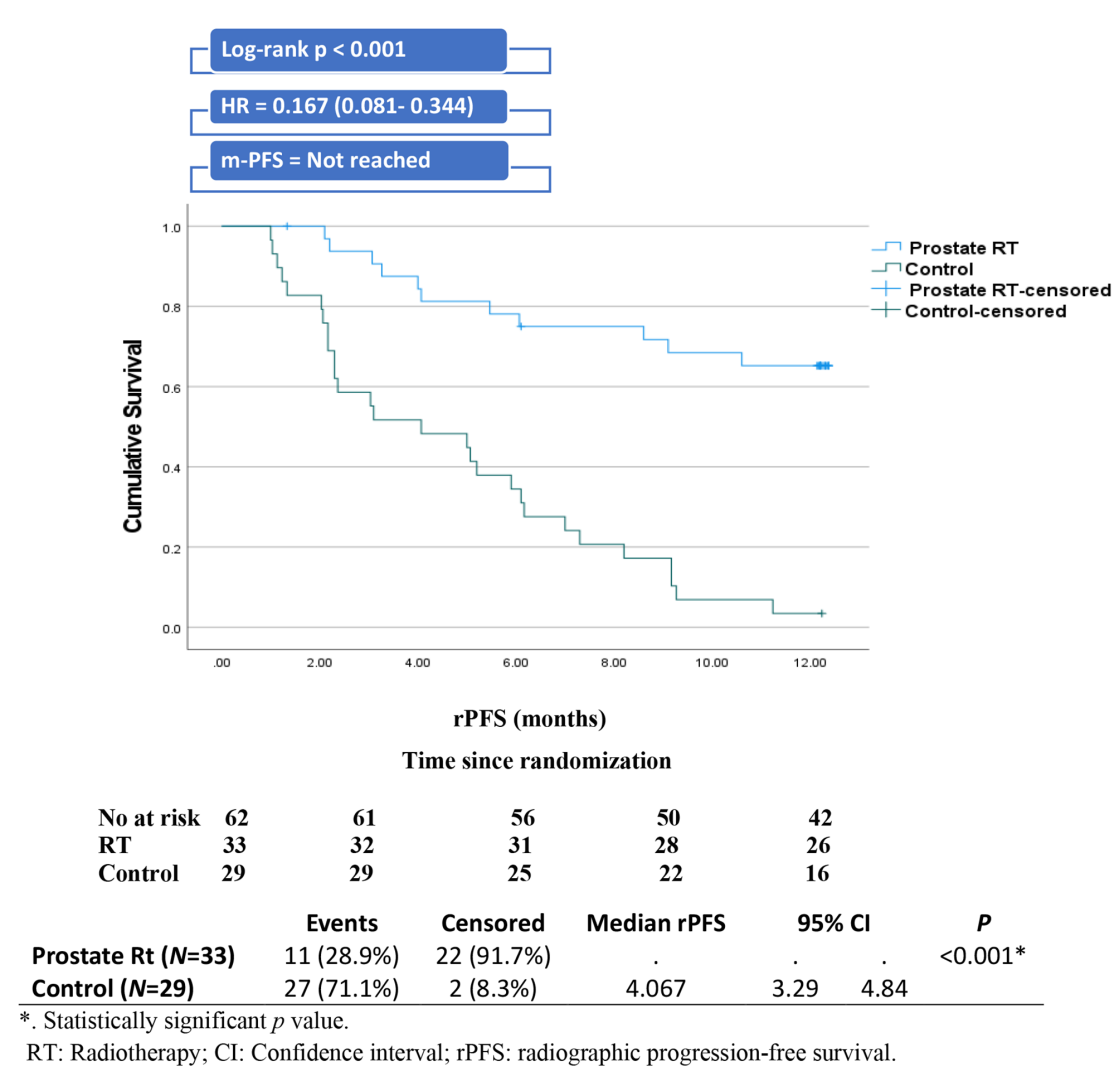
Kaplan-Meir curve of radiographic progression-free survival among study groups

For the radiotherapy group, the median radiographic progression-free survival was not reached, compared to 4.067 months for the control group. The radiotherapy group had a significantly longer radiographic progression-free survival than the control group (not reached vs. 4.067 months, Log-rank *p <* 0.001), with a risk of radiographic progression that was 0.167 times that of the control group (HR: 0.167, 95% CI 0.081–0.344; p < 0.001) (Fig. 2).

**Fig. 3:**
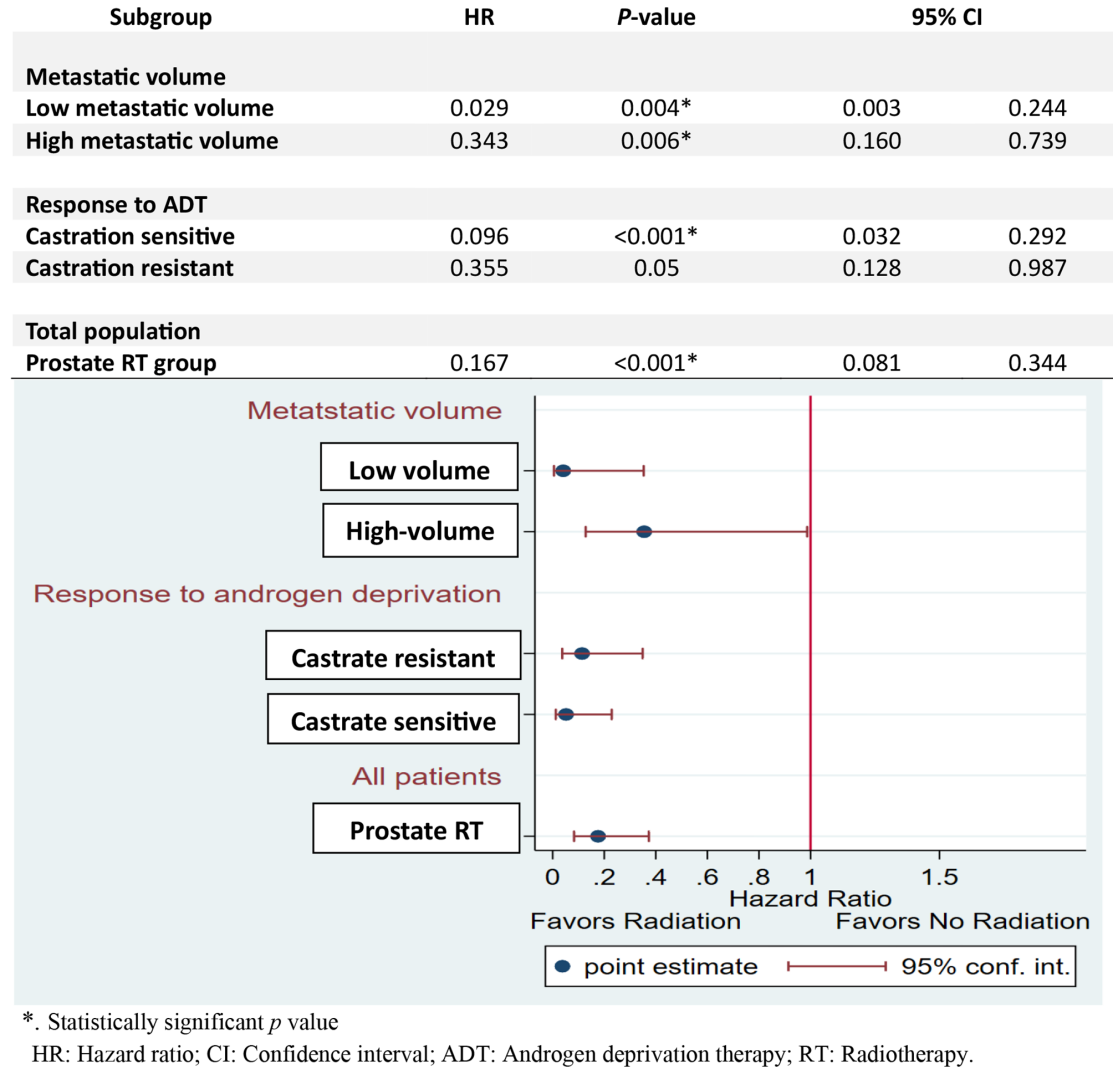
Forest plot for subgroup analysis of progression-free survival

Further confirmatory subgroup analysis revealed that low volume metastatic patients in the radiotherapy group had a significantly lower risk of radiographic progression compared to those in the control group (HR: 0.029, 95% CI 0.003–0.244; p=0.004). Similarly, high-volume metastatic patients in the radiotherapy group had a significantly lower risk of radiographic progression than those in the control group (HR: 0.343, 95% CI 0.160–0.739; p=0.006). Furthermore, the metastatic castration-sensitive patients who received radiotherapy had a significantly lower risk of radiographic progression than those in the control group (HR: 0.096, 95% CI 0.032–0.292; p < 0.001) (Fig. 3).

The multivariable analysis of radiographic progression-free survival in subgroups receiving prostate radiotherapy showed that radiographic progression-free survival was significantly associated with the low volume metastatic subgroup (adjusted HR: 0.104, 95% CI 0.013–0.857; *p* = 0.035) but non significantly associated with the castration-sensitive subgroup (adjusted HR: 0.305, 95% CI 0.083–1.117; *p* = 0.073) or the hypofractionation regimen subgroup (adjusted HR: 0.650, 95% CI 0.185–2.287; *p* = 0.502) (Table 5).

**Table 5:** Univariate and Multivariate subgroup analysis of radiographic progression-free survival in prostate radiotherapy group

Subgroup	Univariate				Multivariate			
	HR	*P-*value	95% CI		HR	*P-*value	95% CI	
			Lower	Upper			Lower	Upper
Metastatic burden								
Low metastatic burden	0.091	0.023*	0.012	0.716	0.104	0.035*	0.013	0.857
High metastatic burden	1				1			
Response to ADT								
Castration sensitive	0.233	0.021*	0.068	0.805	0.305	0.073	0.083	1.117
Castration resistant	1				1			
Fractionation								
Hypofractionation	0.740	0.621	0.226	2.433	0.650	0.502	0.185	2.287
Conventional	1				1			

*. Statistically significant p value

HR: Hazard ratio, CI: Confidence interval, ADT: Androgen deprivation therapy

We conducted a post-hoc analysis which showed that the median radiographic progression-free survival was significantly higher and not reached in the low volume and castration-sensitive metastatic subgroups compared to the high-volume and castration-resistant metastatic subgroups (*p* = 0.004, *p* = 0.012). The risk of radiographic progression among patients in the low volume metastatic subgroup who received prostate radiotherapy was 0.091 times the risk among patients in the high-volume subgroup. No significant difference was found between the two fractionation schedules in terms of median radiographic progression-free survival (*p* = 0.620).

## Discussion

This study was a randomized controlled trial aimed at investigating the benefits of adding prostate radiotherapy to standard androgen deprivation therapy in metastatic prostate cancer patients. We aimed at evaluating the patient-reported health-related quality of life outcomes and the tolerability of prostate radiotherapy in these patients. In this study, we stratified metastatic prostate cancer patients according to their disease burden, classifying them as either having high or low burden metastatic disease. In contrast, Boevé et al. subgrouped their patients based on the number of bone lesions, with < 5, 5–15, or more bone lesions, as determined by bone scan and conventional imaging. They did not stratify patients at the time of randomization [13]. However, Parker et al. also stratified their patients into low and high burden metastatic disease groups based on the metastatic burden at randomization [14].

Prostate radiotherapy was administered to a larger proportion of patients with high-volume metastatic disease in our study compared to Parker et al. and Boevé et al. Our objective was to deliver cytoreductive radiotherapy to their primary prostate with the aim of disrupting the communication pathways between the primary tumor and metastases, impeding the spread of cancer cells to more distant sites, and consequently retarding disease progression. In this subset of patients, we also intended to attain local control of disabling urinary symptoms, thereby positively impacting their health-related quality of life.

With regard to radiation dose, we delivered the same conventionally fractionated regimen of 70 Gy in 35 fractions over 7 weeks, as prescribed by Boevé et al. Alternatively, we delivered the same hypofractionated regimen of 55 Gy in 20 daily fractions over 4 weeks, as prescribed by Parker et al. Apart from the therapeutic benefit, radiobiological rationale, and good local response to the hypofractionated schedule, there were several advantages, such as shortening overall treatment time, saving medical resources, and improving patient convenience [5, 14].

Certainly, an isoeffective hypofractionated treatment regimen (60 Gy/20fx) could be more effective and feasible in metastatic hormone sensitive patients. However, the delivery of this isotoxic escalated dose would mandate the use of more advanced Intensity modulated radiation therapy (IMRT) or Stereotactic ablative body radiotherapy (SABR) techniques to prescribe this isotoxic dose without exceeding the predetermined normal tissue tolerance limits. Unfortunately, these high precision radiotherapy modalities weren’t available in our center.

We preferred using the EORTC QLQ PR-25 as an assessment tool for HRQoL outcome as it focuses on disease specific local disabling symptoms and radiation treatment related adverse events. Several randomized controlled studies also utilized the EORTC QLQ C-30 scale [15, 16]. The EORTC QLQ C-30 includes more generalized, non-symptom specific items on its scale. Our primary concern was to assess local symptoms both before and after radiation therapy and to evaluate radiation treatment effects which were more comprehensively illustrated in EORTC QLQ PR-25 scale.

Monitoring early and late side effects following prostate radiotherapy and their impact on patient’s quality of life is essential for understanding HRQoL. Our results showed that the HRQoL of our patients seems to be at a high level including urinary and bowel functions except for sexual functioning. However, the deterioration in sexual activity and functioning which developed after a time could be a negative consequence of long-term ADT. In contrast, Boevé et al. concluded that patients reported significantly more diarrhea, bowel symptoms, and urinary symptoms after receiving prostate radiotherapy and ADT compared to ADT alone (*p* < 0.001) [17]. Corresponding to our findings, Parker et al. reported that prostate radiotherapy did not negatively impact HRQoL, with no differences in quality-of-life scores over time [6].

Regarding acute and late toxicities, our data showed that acute significant G3 or higher (G3+) genitourinary adverse events were detected in 32.3% of patients in the prostate radiotherapy group. Additionally, acute significant G3 + gastrointestinal adverse events occurred in 14.7% of patients after radiotherapy. On the other hand, late significant G3 + genitourinary adverse events were detected in 12.9%, whereas late significant G3 + gastrointestinal adverse events were seen in 3.2% of patients after radiotherapy. Our study reported more significant G3 + acute and late genitourinary and acute gastrointestinal adverse events than reported by Parker et al. The late significant G3 + genitourinary toxicity was observed in 2% of their patients within 1 year of prostate radiotherapy. Late significant G3 + gastrointestinal toxicities were comparably reported for 3% of their patients [6]. This can be attributed to more patients receiving systemic treatment and the absence of IMRT technique in our centre. Contrary to our study, in which all patients were treated with 3D conformal radiotherapy, Cho et al. reported that 71% of their patients were treated with IMRT, and none of them developed significant gastrointestinal or genitourinary toxicity [18].

The effect of prostate radiotherapy on sexuality exhibited considerable variability among our patients. While some men experienced a complete loss of erectile function, others encountered milder or temporary difficulties. This variability could be attributed to factors such as age, baseline sexual function and comorbidities.

Several randomized controlled trials have consistently shown that cytoreductive prostate radiotherapy, especially when combined with androgen deprivation therapy (ADT), can result in erectile dysfunction for a significant portion of patients. The severity of sexual dysfunction may fluctuate, yet it tends to be more pronounced in patients receiving higher radiation doses and undergoing prolonged ADT durations [19].

Furthermore, the regression analysis demonstrated the significant impact of delivering prostate radiotherapy on radiographic progression-free survival in metastatic prostate cancer patients. Consistent with our findings, Boevé et al. showed that the median progression-free survival was statistically significantly associated with low metastatic volume disease (HR: 0.15, *p* = 0.005) and castration-sensitive disease (HR: 0.22, *p* = 0.004) [20].

Our prespecified subgroup analysis revealed that prostate radiotherapy improved progression-free survival in low metastatic volume (HR: 0.029, *p* = 0.004), high metastatic volume (HR: 0.343, *p* = 0.006), and metastatic castration-sensitive disease (HR: 0.096, *p* < 0.001). Meanwhile, Parker et al. found that prostate radiotherapy improved failure-free survival in all patients (HR: 0.76, *p* < 0.0001) and progression-free survival in patients with low metastatic burden (HR: 0.78, *p* < 0.0001) after a median follow-up of 37 months [14]. Parallel to our data, Parker et al. reported that symptomatic local progression was improved with prostate radiotherapy in the low metastatic burden group [6]. However, Burdett et al. showed that there was no improvement in progression-free survival with prostate radiotherapy [21].

Regarding palliative bone-directed radiotherapy, we treated symptomatic patients similarly to the ORIOLE study conducted by Phillips et al. which demonstrated the clinical and survival benefit of additional treatment of all oligometastases [22]. Likewise, Cho et al. and Morgan et al. also permitted oligometastases directed radiotherapy in addition to prostate radiotherapy [18, 23]. This contradicts Parker et al. and Boevé et al., who did not consider palliative bone-directed radiotherapy [13, 14]. Currently, ESTRO recommends metastases-directed radiotherapy strategies and focuses on the role of combination treatments in oligometastatic prostate cancer patients, as declared by Zilli et al. [24] .

There were some limitations in our study. First, the small sample size may affect its external validity. Second, the lack of highly advanced precision radiation techniques such as IMRT in our center was an important limitation. Besides, we did not require the use of PSMA-PET due to limited resources in our center, although PSMA-PET is more accurate.

and precise in initial staging than conventional imaging [25, 26]. Additionally, the presence of missing data could pose a challenge, potentially introducing bias into our analysis. Lastly, it’s worth noting that HRQoL assessments frequently depend on self-reported data from patients. This subjectivity could introduce bias, as patients might interpret and respond to questions differently based on their personal experiences and perceptions.

## Conclusion

In conclusion, the administration of prostate radiotherapy to metastatic prostate cancer patients can improve patient-reported health-related quality of life outcomes, particularly urinary function with bowel function preservation. The HRQoL after prostate radiotherapy in metastatic prostate cancer patients correlates with the total delivered dose, fractionation regimen and beam delivery techniques. Most acute radiation-induced toxicities were transient and manageable without significant long-term effects. We recommend that this study can be replicated with a larger sample size and longer follow-up duration to assess potential late effects in patients who received prostate radiotherapy and their impact on HRQoL.

Understanding the quality of life in metastatic prostate cancer patients after prostate radiotherapy is vital for healthcare providers. It helps them make informed shared decisions about treatment options, manage side effects, and provide support services that address the specific needs and concerns of each patient, ultimately improving their overall well-being and survivorship. Additionally, novel studies are awaited to test the long-term health-related quality of life outcomes of combination therapies as part of a treatment intensification plan for metastatic hormonal-sensitive prostate cancer patients.

### Electronic supplementary material

Below is the link to the electronic supplementary material.

Supplementary Material 1

Supplementary Material 2

## Data Availability

Data supporting this research findings were collected from registries and medical records at the Department of Clinical Oncology, Suez Canal University Hospital. The study protocol and datasets are attached and included within the article.

## Abbreviations

HRQoL: Health-related quality of life
PROs: Patient-reported outcomes
ADT: Androgen deprivation therapy
EORTC QLQ-PR25: European Organization for Research and Treatment of Cancer Quality of Life Questionnaire
ESTRO: European Society for Radiotherapy and Oncology
rPFS: Radiographic progression-free survival
PROMs: Patient-reported outcomes measures
ECOG: Eastern Cooperative Oncology Group
RT: Radiotherapy
G2: Grade 2
G3+: Grade 3 or higher
IMRT: Intensity-modulated radiation therapy
SABR: Stereotactic ablative body radiotherapy

## References

[1] Siegel RL, Miller KD, Wagle NS, Jemal A, Cancer statistics. 2023. CA Cancer J Clin. 2023;73(1):17–48. 10.3322/caac.21763. 10.3322/caac.21763 36633525

[2] Siegel DA, O’Neil ME, Richards TB, Dowling NF, Weir HK (2020). Prostate Cancer incidence and survival, by stage and Race/Ethnicity — United States, 2001–2017. MMWR Morb Mortal Wkly Rep.

[3] Menges D, Yebyo HG, Sivec-Muniz S, Haile SR, Barbier MC, Tomonaga Y et al. Treatments for Metastatic Hormone-sensitive Prostate Cancer: Systematic Review, Network Meta-analysis, and Benefit-harm assessment. Eur Urol Oncol. 2022;5(6):605–16. Available from: https://www.sciencedirect.com/science/article/pii/S2588931122000657. 10.1016/j.euo.2022.04.007 35599144

[4] Houédé N, Rébillard X, Bouvet S, Kabani S, Fabbro-Peray P, Trétarre B et al. Impact on quality of life 3 years after diagnosis of prostate cancer patients below 75 at diagnosis: An observational case-control study. BMC Cancer. 2020;20(1):757. 10.1186/s12885-020-07244-y. 10.1186/s12885-020-07244-y PMC7424648 32787797

[5] Boevé LMS, Hulshof MCCM, Vis AN, Zwinderman AH, Twisk JWR, Witjes WPJ (2019). Effect on survival of androgen deprivation therapy alone compared to androgen deprivation therapy combined with concurrent radiation therapy to the prostate in patients with primary bone metastatic prostate cancer in a prospective randomised clinical tria. Eur Urol.

[6] Parker CC, James ND, Brawley CD, Clarke NW, Ali A, Amos CL et al. Radiotherapy to the prostate for men with metastatic prostate cancer in the UK and Switzerland: Long-term results from the STAMPEDE randomised controlled trial. PLoS Med. 2022;19(6):e1003998. 10.1371/journal.pmed.1003998. 10.1371/journal.pmed.1003998 PMC9173627 35671327

[7] Nakamura K, Konishi K, Komatsu T, Ishiba R. Quality of life after external beam radiotherapy for localized prostate cancer: Comparison with other modalities. Int J Urol. 2019;26(10):950–4. 10.1111/iju.14026. 10.1111/iju.14026 31131492

[8] Mallick S, Madan R, Julka PK, Rath GK (2015). Radiation induced cystitis and proctitis - prediction, assessment and management. Asian Pac J Cancer Prev.

[9] Briggs LG, Sentana-Lledo D, Lage DE, Trinh QD, Morgans AK. Optimal assessment of quality of life for patients with prostate cancer. Ther Adv Med Oncol. 2022;14:17588359221141306. 10.1177/17588359221141306. 10.1177/17588359221141306 PMC9747880 36531831

[10] van Andel G, Bottomley A, Fosså SD, Efficace F, Coens C, Guerif S (2008). An international field study of the EORTC QLQ-PR25: a questionnaire for assessing the health-related quality of life of patients with prostate cancer. Eur J Cancer.

[11] Guckenberger M, Lievens Y, Bouma AB, Collette L, Dekker A, deSouza NM et al. Characterisation and classification of oligometastatic disease: a European Society for Radiotherapy and Oncology and European Organisation for Research and Treatment of Cancer consensus recommendation. Lancet Oncol [Internet]. 2020;21(1):e18–28. 10.1016/S1470-2045(19)30718-1. 10.1016/S1470-2045(19)30718-1 31908301

[12] Kuhl CK, Alparslan Y, Schmoee J, Sequeira B, Keulers A, Brümmendorf TH et al. Validity of RECIST version 1.1 for response assessment in metastatic cancer: A prospective, multireader study. Radiology. 2019;290(3):349–56. 10.1148/radiol.2018180648. 10.1148/radiol.2018180648 30398433

[13] Boevé L, Hulshof M, Vis A, Zwinderman K, Twisk J, Delaere K (2018). Pd10-10 a prospective, randomized controlled trial evaluating overall survival in patients with primary bone metastatic prostate Cancer (Mpca) receiving either androgen deprivation therapy (adt) or Adt Combined with Concurrent Radiation Therapy to the pro. J Urol.

[14] Parker CC, James ND, Brawley CD, Clarke NW, Hoyle AP, Ali A (2018). Systemic therapy for Advanced or metastatic prostate cancer: evaluation of drug efficacy (STAMPEDE) investigators. Radiotherapy to the primary tumour for newly diagnosed, metastatic prostate cancer (STAMPEDE): a randomised controlled phase 3 trial. Lancet.

[15] Warde P, Mason M, Ding K, Kirkbride P, Brundage M, Cowan R (2011). Combined androgen deprivation therapy and radiation therapy for locally advanced prostate cancer: a randomised, phase 3 trial. Lancet.

[16] Adam S, Koch-Gallenkamp L, Bertram H, Eberle A, Holleczek B, Pritzkuleit R et al. Health-related quality of life in long-term survivors with localised prostate cancer by therapy—Results from a population-based study. Eur J Cancer Care (Engl). 2019;28(5):e13076. 10.1111/ecc.13076. 10.1111/ecc.13076 31050091

[17] Boevé L, Hulshof MCCM, Verhagen PCMS, Twisk JWR, Witjes WPJ, de Vries P et al. Patient-reported Quality of Life in Patients with Primary Metastatic Prostate Cancer Treated with Androgen Deprivation Therapy with and Without Concurrent Radiation Therapy to the Prostate in a Prospective Randomised Clinical Trial; Data from the HORRAD T. Eur Urol. 2021;79(2):188–97. Available from: https://www.sciencedirect.com/science/article/pii/S0302283820306400. 10.1016/j.eururo.2020.08.023 32978014

[18] Cho Y, Chang JS, Rha KH, Hong SJ, Choi YD, Ham WS (2016). Does radiotherapy for the primary tumor benefit prostate cancer patients with distant metastasis at initial diagnosis?. PLoS ONE.

[19] Gryzinski GM, Fustok J, Raheem OA, Bernie HL (2022). Sexual function in men undergoing androgen deprivation therapy. Androg Clin Res Ther.

[20] Gregucci F, Fiorentino A, Re: Liselotte MS, Boevé, Maarten CCM, Hulshof AndréN, Vis (2019). Effect on survival of androgen deprivation therapy alone compared to Androgen Deprivation Therapy Combined with Concurrent Radiation Therapy to the prostate in patients with primary Bo. Eur Urol.

[21] Burdett S, Boevé LM, Ingleby FC, Fisher DJ, Rydzewska LH, Vale CL (2019). Prostate radiotherapy for metastatic hormone-sensitive prostate Cancer: a STOPCAP systematic review and Meta-analysis. Eur Urol.

[22] Phillips R, Shi WY, Deek M, Radwan N, Lim SJ, Antonarakis ES et al. Outcomes of Observation vs Stereotactic Ablative Radiation for Oligometastatic Prostate Cancer: The ORIOLE Phase 2 Randomized Clinical Trial. JAMA Oncol. 2020;6(5):650–9. 10.1001/jamaoncol.2020.0147. 10.1001/jamaoncol.2020.0147 PMC7225913 32215577

[23] Morgan SC, Holmes OE, Craig J, Grimes S, Malone S. Long-term outcomes of prostate radiotherapy for newly-diagnosed metastatic prostate cancer. Prostate Cancer Prostatic Dis. 2021;24(4):1041–7. 10.1038/s41391-021-00339-y. 10.1038/s41391-021-00339-y 33820949

[24] Zilli T, Achard V, Dal Pra A, Schmidt-Hegemann N, Jereczek-Fossa BA, Lancia A et al. Recommendations for radiation therapy in oligometastatic prostate cancer: An ESTRO-ACROP Delphi consensus. Radiother Oncol. 2022;176:199–207. 10.1016/j.radonc.2022.10.005. 10.1016/j.radonc.2022.10.005 36228761

[25] Roberts MJ, Maurer T, Perera M, Eiber M, Hope TA, Ost P et al. Using PSMA imaging for prognostication in localized and advanced prostate cancer. Nat Rev Urol. 2022;1–25. 10.1038/s41585-022-00670-6 36473945

[26] Galgano SJ, McDonald AM, West JT, Rais-Bahrami S (2022). Defining Oligometastatic Disease in the New era of PSMA-PET imaging for primary staging of prostate Cancer. Cancers (Basel).

