# Correction: Health-related quality of life of metastatic prostate cancer patients treated with prostate Radiotherapy

**DOI:** 10.1186/s12885-024-12217-6

**Published:** 2024-04-05

**Authors:** Heba Maged Ayoub, Maha Lotfy Zamzam, Eman Essam Elsemary, Ihab Mohamed Hassanin, Fifi Mostafa Elsayed

**Affiliations:** Clinical Oncology Department, Faculty of Medicine, Suez Canal University, Ismailia, Egypt

**Correction: BMC Cancer 23, 927 (2023)**

10.1186/s12885-023-11448-3

Following publication of the original article [1], the authors identified an error in the equal contributions statement. The statement should read as follows: † Heba Maged Ayoub, Maha Lotfy Zamzam, Eman Essam Elsemary, Ihab Mohamed Hassanin and Fifi Mostafa Elsayed contributed equally to this work.

The original article [1] has been corrected.
