# Supplementary figures and images for: Health-related quality of life of metastatic prostate cancer patients treated with prostate Radiotherapy
Source: BMC Cancer. 2023 Oct 2;23:927. doi: 10.1186/s12885-023-11448-3 (PMC10544568; doi:10.1186/s12885-023-11448-3)

**
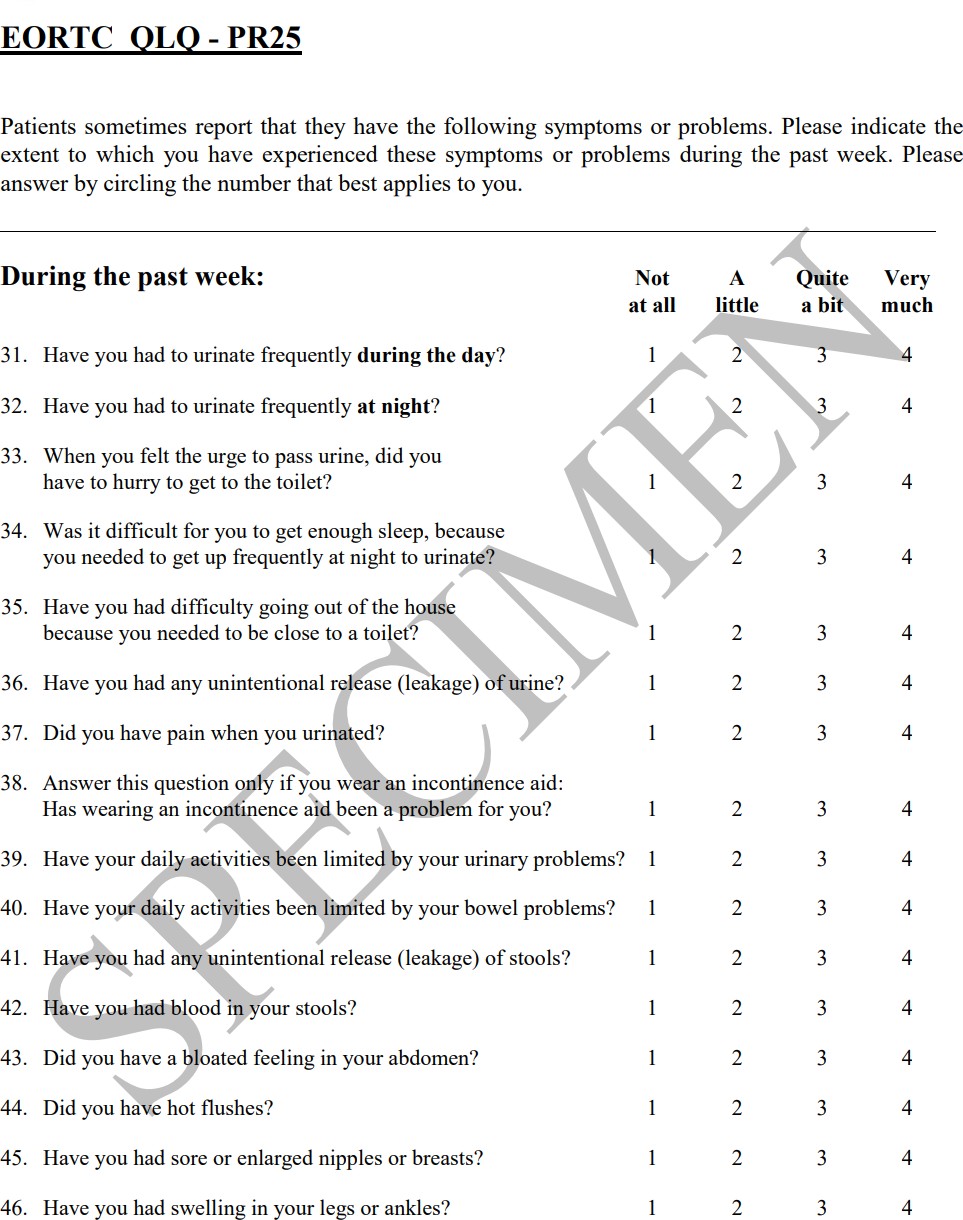
Supplementary File 1.** **EORTC QLQ-PR25**


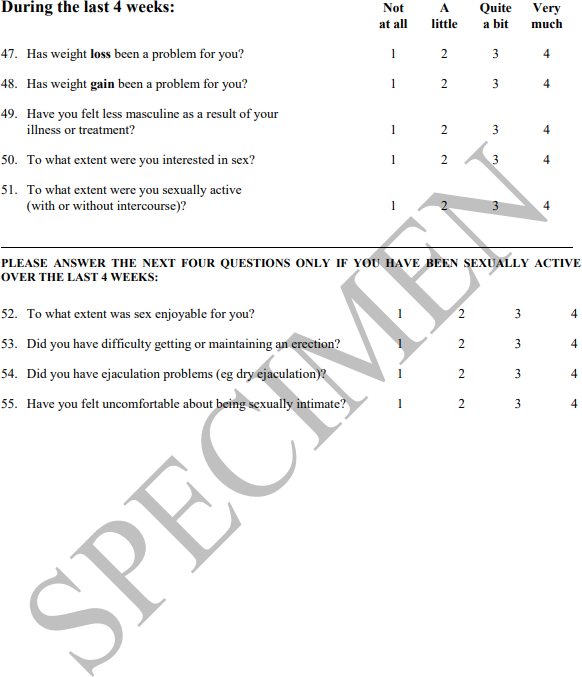

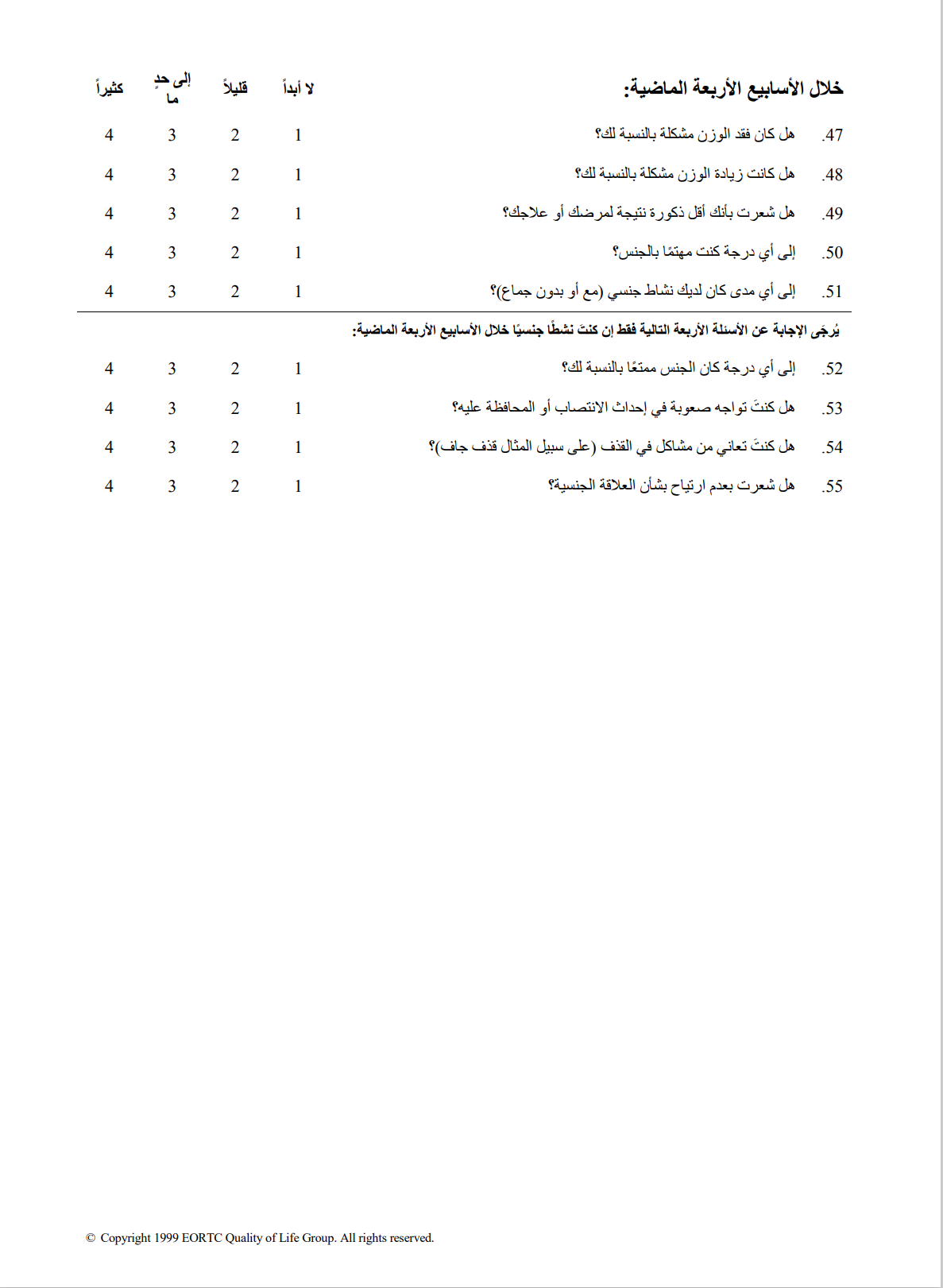

Supplement: Supplementary file 1 — Supplementary Material 1 [file 12885_2023_11448_MOESM1_ESM.docx]

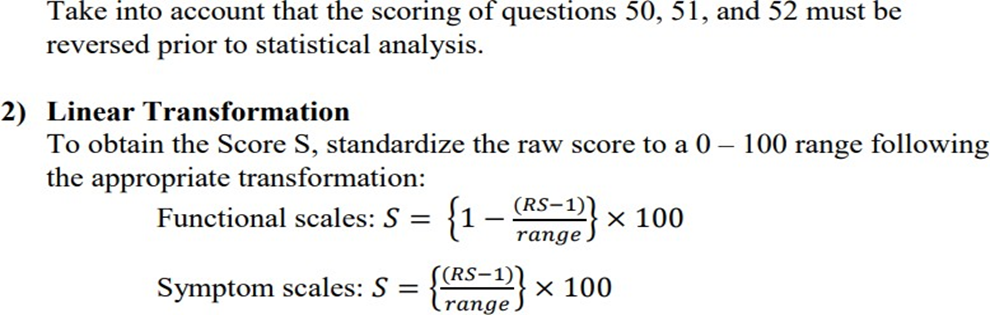

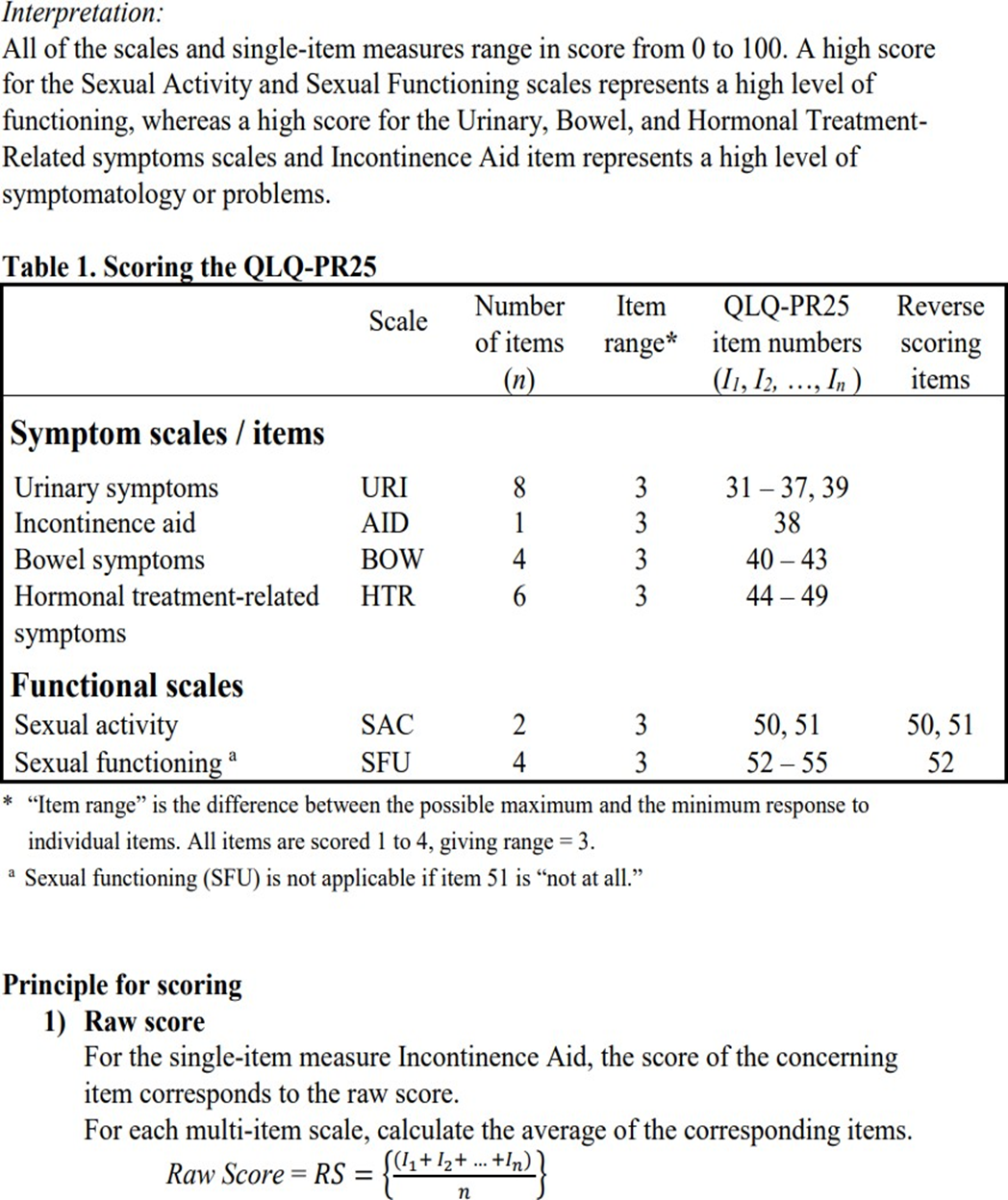

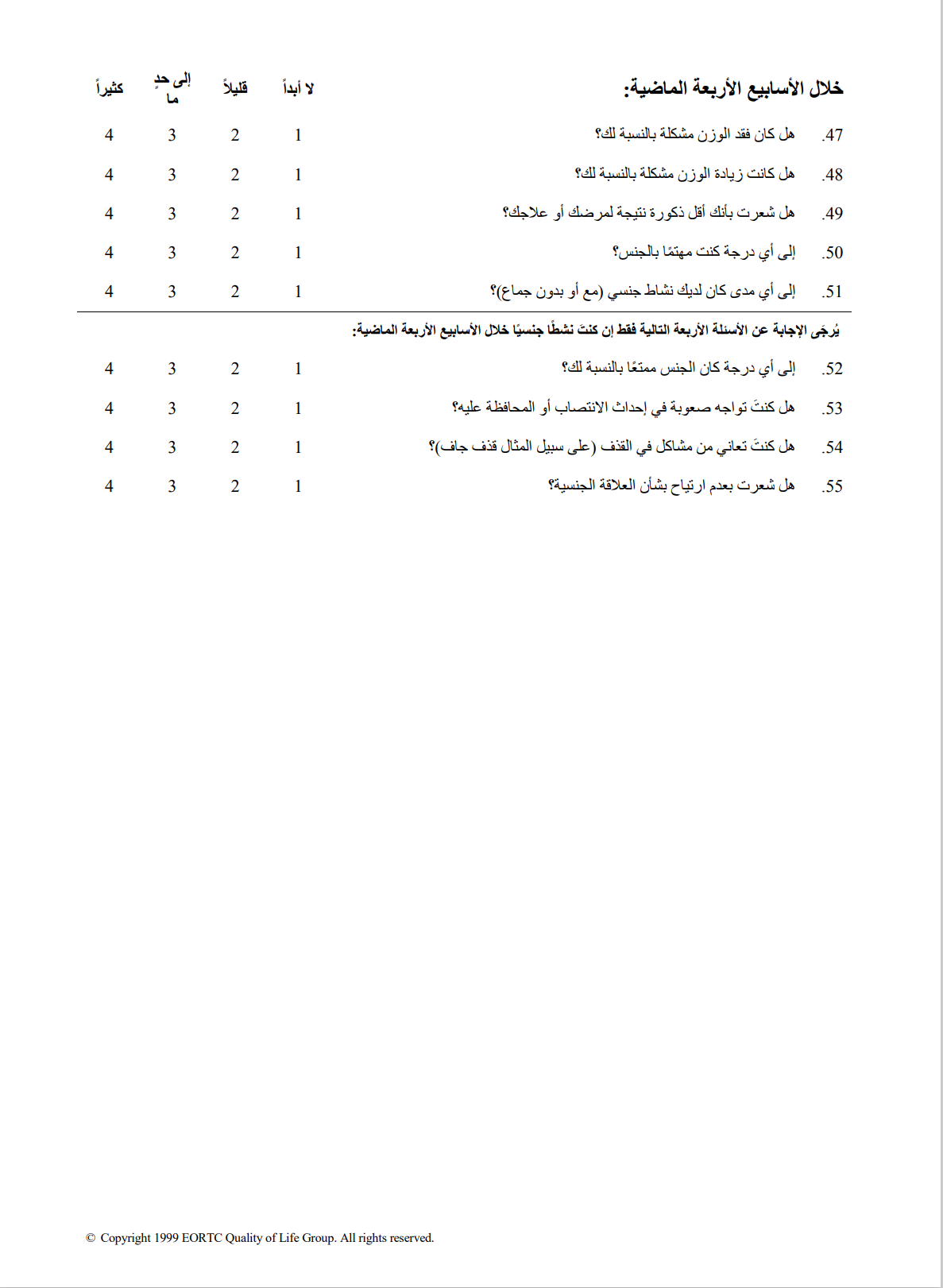
**Supplementary File 2.** **EORTC QLQ-PR25 Interpretation**

Supplement: Supplementary file 2 — Supplementary Material 2 [file 12885_2023_11448_MOESM2_ESM.docx]
